# A patient with triple-negative breast cancer developed acute myocardial infarction after docetaxel chemotherapy: case report and literature review

**DOI:** 10.3389/fcvm.2026.1791074

**Published:** 2026-05-29

**Authors:** Fangqin Tong, Zhipeng Fan, Huiling Huang, Chen Chen, Jingzhou Xiang, Na Li, Min Xia, Lei Li

**Affiliations:** 1 Department of Oncology, Wuhan Asia General Hospital, Wuhan, China; 2 Pharmacy Department, Wuhan Asia General Hospital, Wuhan, China

**Keywords:** acute myocardial infarction, breast cancer, cardio-oncology, docetaxel, drug adverse reactions, paclitaxel

## Abstract

We report the first case of an acute ST-segment elevation myocardial infarction (AMI) induced by docetaxel in a patient with triple-negative breast cancer (BC). Coronary angiography showed severe occlusions in multiple coronary arteries, and timely PCI surgery was successfully performed, saving the patient's life. Docetaxel is a commonly used taxane chemotherapy drug for treating breast cancer, which can cause numerous adverse drug reactions in cancer treatment. However, compared with paclitaxel, reports of docetaxel-induced cardiotoxicity are fewer and generally milder, and acute myocardial infarction induced by docetaxel is extremely rare and potentially fatal when it occurs. Due to this severe adverse drug reaction, the subsequent docetaxel chemotherapy regimen was discontinued. In addition, we review this case alongside 12 published case reports and conduct a comprehensive analysis of 94 reported instances of myocardial infarction associated with taxane use from the FDA Adverse Event Reporting System (FAERS) database, evaluating their clinical relevance and key characteristics. In this case, we highly suspect that docetaxel directly caused the acute myocardial infarction, and propose that the mechanism may involve coronary vasospasm, endothelial dysfunction, or prothrombotic effects, although the exact pathway remains unclear. This study, through the rare case of a breast cancer patient who experienced acute myocardial infarction after docetaxel chemotherapy, demonstrates timely and proper cardiac management in a patient assessed as low-risk for cardiotoxicity with no relevant cardiovascular factors. Furthermore, through literature review combined with the FAERS database, we further discuss possible mechanisms, influencing factors, and predictive methods to provide clinical reference and warnings.

## 1 Introduction

Breast cancer (BC) is the most common cancer among women and a leading cause of cancer-related deaths (1). Docetaxel is a commonly prescribed taxane cytotoxic antitumor drug approved by the U. S. Food and Drug Administration for treating several cancers, including breast, head and neck, lung, and prostate cancer (2). Multiple reports from clinical trials, case studies, and the FDA Adverse Event Reporting System have documented serious, sometimes life-threatening, side effects of docetaxel. Its frequent adverse effects include myelosuppression, alopecia, cardiotoxicity, fluid retention, and allergic reactions (3). Combining docetaxel with other chemotherapy drugs can amplify the severity of its side effects. Anthracyclines like doxorubicin and epirubicin are often co-administered for breast cancer, greatly enhancing disease-specific survival (4). However, these regimens are linked to cardiovascular side effects and a higher risk of cardiovascular mortality. Many publications have clearly identified cardiotoxicity as a major adverse effect of anthracycline chemotherapy (5–10). Notably, our patient underwent four cycles of epirubicin with cyclophosphamide without any cardiac side effects, yet she suffered a severe, extensive anterior myocardial infarction just 12 h after her initial docetaxel monotherapy cycle. We must recognize that we underestimated the cardiotoxic potential of docetaxel and the cumulative toxicity of sequential chemotherapy regimens. Here, we present a rare case of acute myocardial infarction during docetaxel therapy for breast cancer. Although the patient was promptly identified and transferred to intensive care for emergency intervention, this case provides a critical clinical alert. Through a systematic literature review and an analysis of 94 cases of taxane-associated acute myocardial infarction reported in the FAERS database from 2004 to the third quarter of 2025, we aim to define the clinical features, mechanisms, and contributing factors behind these events. This analysis helps balance the benefits and risks of related chemotherapy regimens.

## 2 Case presentation

### 2.1 Clinical case presentation

A 67-year-old woman presented to our hospital in February 2025 with a left breast mass. A breast ultrasound and biopsy confirmed invasive carcinoma of the left breast with no detectable metastases. She subsequently underwent a left-sided mastectomy with sentinel lymph node and axillary dissection. Postoperative pathology diagnosed invasive carcinoma of the left breast (T2N1M0 stage IIB, estrogen receptor: negative, progesterone receptor: negative, HER2: 1 + negative), Ki-67 (60%). Upon admission, all blood tests and electrolytes were within normal limits. She had her first period at 13 and has now reached menopause. No history of hypertension, hyperlipidaemia, coronary heart disease or other cardiovascular risk factors,and no drug allergy history. The patient was classified as low-risk according to the SCORE2 risk assessment (47).

Pre-therapy echocardiogram showed no left ventricular enlargement, normal ejection fraction (60%), no valve disease, and no pericardial effusion. Her electrocardiogram was normal (Figure 1A). Chemotherapy began in March 2025: four cycles of epirubicin (100 mg/m^2^) and cyclophosphamide (600 mg/m^2^). During treatment, the patient had mild myelosuppression but no other side effects. The fifth cycle comprised docetaxel monotherapy (75 mg/m^2^) with dexamethasone, diphenhydramine, and ondansetron as premedications. She reported no discomfort during infusion. At 9:30 AM the following day, she developed chest pain, dyspnea, profuse sweating, and subsequently, pain radiating to her left arm and back. Blood pressure was 165/95 mmHg; heart rate 112bpm. Nitroglycerin provided little improvement. Blood tests evaluated cardiac enzymes and other markers. A bedside ECG showed sinus rhythm with abnormal ST-T changes in the left atrium (Figure 1B). Cardiac enzyme levels were normal during initial symptoms. Two hours later, repeat tests showed a markedly elevated high-sensitivity troponin level of 16,372. 00 ng/L (reference: <45. 2 ng/mL) (Figure 2). A follow-up ECG revealed an inferior and anterior wall myocardial infarction with significant ST-segment elevation in leads V1-5(Figure 1C). Cardiology diagnosed an acute inferior and anterior wall myocardial infarction, and she was promptly transferred to the cardiac intensive care unit (CCU). Emergency coronary angiography found 75% stenosis of the right coronary artery; Complete occlusion of the proximal left anterior descending artery, as well as 70% stenosis of the distal circumflex artery and the first obtuse marginal branch (Figure 3). Emergent percutaneous coronary intervention (PCI) was performed. Afterward, she noted marked relief of her chest tightness. However, troponin levels remained high, signaling severe myocardial injury and risk of cardiac rupture. Troponin gradually declined. Following PCI, she maintained anticoagulant therapy with enoxaparin sodium.Subsequent cardiac markers normalized, symptoms abated, and she was discharged from cardiology. Multiple follow-up assessments, including clinical evaluation and echocardiography, remained normal. Pre-chemotherapy laboratory tests revealed a normal lipid profile and a slightly elevated D-dimer (1.09 µg/mL). Based on the assessment by the cardiology team, the presence of multi-vessel coronary occlusion suggested that acute myocardial infarction was most likely attributable to *in situ* thrombus formation, with embolism secondary to plaque detachmen deemed unlikely.

**Figure 1:**
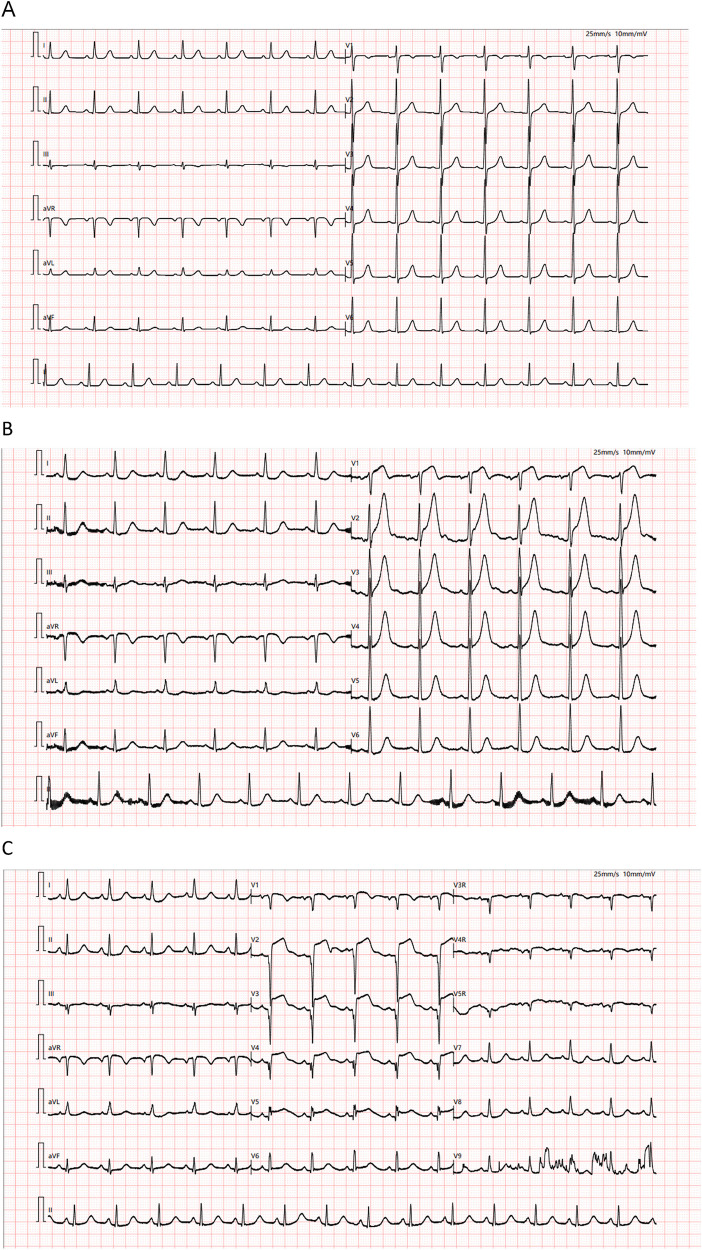
Figure **(A)** is the normal ECG before docetaxel treatment; figure **(B)** is the ECG during the onset of myocardial infarction symptoms; figure **(C)** shows the ECG 2 h after symptom onset.

**Figure 2:**
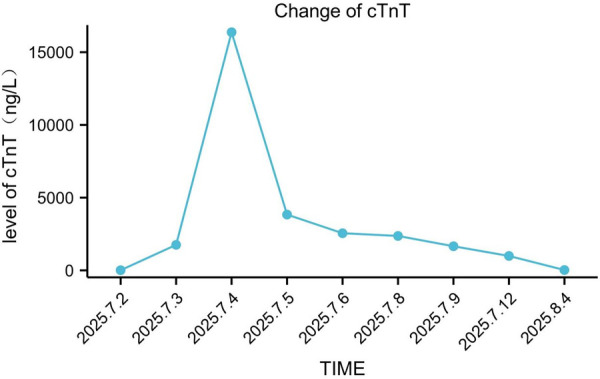
The process of high-sensitivity cardiac troponin T changes from the onset of symptoms.

**Figure 3:**
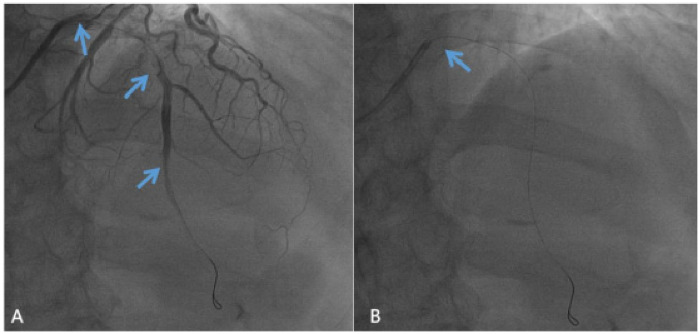
Figure **A**: 100% stenosis in the proximal segment of the left anterior descending artery; and 70% stenosis in the distal segment of the circumflex artery and the first obtuse marginal branch. Figure **B**: Coronary angiography shows 75% stenosis in the right coronary artery.

### 2.2 Literature search of paclitaxel-induced myocardial infarction

We searched the PubMed and Google Scholar databases for published studies on paclitaxel-induced myocardial infarction through 15 December 2025 using the keywords “paclitaxel”, “Docetaxel”, “myocardial infarction”, and “acute coronary syndrome”. There were 12 cases, which are summarized in Table 1.

**Table 1:** Literature search on paclitaxel-induced myocardial infarction.

Country	Author/Year	Gender	Age	Cancer type	Cardiovascular risk factors	Medication and treatment cycle	Pre-chemotherapy treatment	Timing of onset	Symptoms	ECG	Myocardial enzyme	Coronary angiogram	Management	Outcome
India	Shah K et al. (11)（2012）	F	45	Ovarian cancer	None	PTX/First	Corticosteroid medication	3h after start of infusion	Chest pain, dyspnea，sweating	ST segment depression in V1, V2, and AVL	Positive	Not done	Medical management	Death
U. K	S. Laher et al. (12)（1997）	F	61	Ovarian cancer	None	PTX/First	Corticosteroid medication	6h after start of infusion	Chest pain, dyspnea	ST elevation in V1-V6	Positive	Not done	Medical management	Death
U. S	Elham Hekmat et al. (13)（1996）	F	67	breast cancer	Not mentioned	PTX/unknown	Corticosteroid medication	15h after completion of infusion	Chest pain, dyspnea	ST elevation in II, III, aVF	Positive	Not done	unknown	Alive
Japan	Shota Higami et al. (14)（2022）	F	48	Cervical cancer	None	PTX/First	unknown	After completion of infusion	sweating，Unconscious	ST elevation in I, II, III, aVL, aVF, V4-V6	Positive	Normal	unknown	Alive
South Korea	Sang-Ho Park et al. (15)（2009）	F	63	Ovarian cancer	Hypertension	PTX/First	unknown	Day after completion of infusion	Typical angina	ST elevation in V2-V5	Positive	LM + dLAD	Medical management	Alive
Germany	Carsten Schrader et al. (16)（2005）	F	58	Ovarian cancer	None	PTX/First	Corticosteroid medication	During the infusion process	Chest pain, nausea	ST elevation in II, III, aVF	Normal	Not done	Transferred to the ICU	Alive
Turkey	Gökmen Gemici et al. (17)（2009）	F	51	Ovarian cancer	Myocardial infarction, PCI	PTX/Second	unknown	Minutes after start of infusion	Chest pain, sweating	ST elevation in II, III, aVF, V3-V6	Positive	80% LCx	Stent placement	Alive
India	Vikram A Londhey et al. (18)（2009）	F	48	Ovarian cancer	None	PTX/Fifth	unknown	After completion of infusion	Circulatory collapse	ST elevation in V2	unknown	Not done	Medical management	Death
U. S	Christopher Esber et al. (19)（2014）	F	47	breast cancer	None	PTX/Second	unknown	Minutes after start of infusion	Ventricular fibrillation	ST elevation in V1-V3	unknown	pLAD + vasospasm in m-/d-LAD	Stent placement	Alive
India	Gautam Rawal et al. (20)（2016）	M	63	Esophageal cancer	None	PTX/unknown	unknown	After completion of infusion	Chest pain, sweating	ST elevation in II, III, aVF	Positive	100% RCA	Stent placement	Alive
Qatar	Gi Eun Kim et al. (21)（2024）	M	71	Lung adenocarcinoma	Hypertension, smoking，PCI	PTX/Fifth	Corticosteroid medication	During the infusion process	Chest pain, dyspnea	The ST segment of the inferior leads is depressed.	Positive	80%LAD distal，100%RCA prox	Stent placement	Alive
China	Huizhu Liang et al. (22)（2021）	M	62	Bladder cancer	Hypertension, smoking	PTX/unknown	unknown	Minutes after start of infusion	Chest pain, dyspnea	ST elevation in II, III, aVF	unknown	90%RCA prox	Medical management	Alive

LAD, Left anterior descending artery; LM, Left main artery; LCX, Left circumflex artery; RCA, Right coronary artery; PCI, Percutaneous coronary intervention; PTX:Paclitaxel; DTX, Docetaxel.

### 2.3 Analysis of paclitaxel-induced myocardial infarction cases in the FAERS database

The FAERS database identified 94 cases of acute myocardial infarction associated with paclitaxel use, spanning 2004 to the third quarter of 2025 (Table 2). Among the 94 included cases, 56 were reported by healthcare professionals and 7 by consumers. With respect to gender distribution, 1 case had undetermined gender, while 58 cases were male and 35 were female. After excluding cases with missing weight data, the largest subgroup (31 cases) consisted of individuals weighing 50–100 kg. Age information was unavailable in 10 cases. Of the remaining cases, 32 were aged 18–64 years and 52 were aged 65–85 years. Clinical outcomes comprised 22 fatal cases (23%), 19 life-threatening cases (20%), yielding an overall total of 67 serious cases (71%).

**Table 2:** Clinical data of 94 paclitaxel-related acute myocardial infarction in the FAERS database.

Characteristics	Value (*n* = 94)
Gender	
Male	58 (61. 7%)
Female	35 (37. 2%)
Missing	1 (1. 1%)
Weight (kg)	
<50	1 (1. 1%)
50–100	31 (33. 0%)
>100	10 (10. 6%)
Missing	52 (55. 3%)
Age (years)	
18–64	32 (34. 0%)
65–85	52 (55. 3%)
Missing Outcome	10 (10. 6%)
Death	22 (23. 4%)
Hospitalization	21 (22. 3%)
Life-Threatening	19 (20. 2%)
Other Serious	32 (34. 0%)
No	27 (28. 7%)
Yes	67 (71. 3%)
Reporter Type	
Consumer	7 (7. 4%)
Health Professional	17 (18. 1%)
Pharmacist	4 (4. 3%)
Physician	35 (37. 2%)
Missing	31 (33. 0%)

## 3 Discussion

Chronic inflammation in cancer makes patients susceptible to arterial and venous thromboembolism, including myocardial infarction (42). Moreover, chemotherapy itself is increasingly associated with cardiotoxicity. Docetaxel is a paclitaxel-based antitumor drug, while paclitaxel is structurally similar to taxane B, a cardiotoxic alkaloid derived from the taxus plant. Compared to paclitaxel, there are fewer reports of cardiotoxicity with docetaxel, and the severity is milder, with myocardial infarction being extremely rare. Among the 12 retrieved articles, possible mechanisms underlying paclitaxel- induced myocardial infarction were proposed (11, 22). "Allergic myocardial infarction” and Kounis syndrome were also used to explain paclitaxel-related myocardial infarction. The relevant mechanisms suggest that inflammatory mediators, including proteases, trypsin, arachidonic acid products, platelet-activating factor, and cytokines and chemokines, are released during mast cell activation (23, 24). Allergic reactions were observed in 16% to 40% of patients after injection (1). Related studies have shown that the mechanism by which paclitaxel drugs cause myocardial ischemia is through the induction of histamine release and stimulation of H1 and H2 receptors, resulting in increased myocardial oxygen demand, coronary artery vasoconstriction, and time- dependent effects that damage the myocardium (11–13, 15). Esber et al. showed that the drug carrier Cremophor EL polyoxygenated castor oil, when combined with paclitaxel and docetaxel, caused severe allergic reactions, leading to acute myocardial infarction caused by prolonged coronary artery vasoconstriction. Castor oil in the suspension increased histamine and intracellular calcium concentrations (19). Bibera et al. also showed that myocardial infarction may be related to inactive ingredients in drug formulations, especially for paclitaxel and docetaxel, whose excipients (polyoxyethylene castor oil and polysorbate 80) are considered most likely to cause these cardiotoxic reactions (25). It is worth noting that in Haddad's research, to enhance the solubility and pharmacological properties of the drug, a variety of paclitaxel delivery systems were developed. The most commonly used paclitaxel delivery system is the co-solvent strategy of ethanol and Cremophor EL™ (polyoxyethyl castor oil). Studies have shown that although this method overcomes the solubility problem, Cremophor EL remains associated with allergies, neurotoxicity, and nephrotoxicity (26–28). Currently, paclitaxel-coated balloons and stents are commonly used in coronary artery reconstruction (29). In the study of Haffler Z et al., a severe allergic reaction occurred when paclitaxel drug-eluting stents were used for local administration (30). This is contradictory to the myocardial infarction caused by the use of paclitaxel drugs in chemotherapy patients. Whether the use of paclitaxel drug- eluting stents and balloons will further aggravate the occurrence of myocardial infarction requires further research with a large number of clinical cases. Docetaxel is a taxane drug, but there is currently no research supporting whether the co-solvent in its drug delivery system can cause cardiotoxicity and allergic reactions leading to myocardial infarction. Relevant studies indicate that docetaxel has relatively good cardiovascular safety (31), but in the study by Mishra T et al. (32), patients receiving docetaxel chemotherapy experienced severe cerebral ischaemic symptoms, and the exact mechanism has not been elucidated. However, this study indeed lacks sufficient evidence to indicate a direct causal relationship between the use of docetaxel and the occurrence of acute myocardial infarction in this case, though a correlation cannot be ruled out. This case may serve as a warning in clinical cancer treatment.

In this study, only 4 patients had cardiovascular risk factors. Acute myocardial infarction occurred in 50% of patients during the first cycle of chemotherapy (Table 1). Specifically, 10 patients developed acute myocardial infarction within minutes to hours following paclitaxel or docetaxel infusion, while 3 patients experienced the event during drug infusion. On electrocardiography, 11 patients showed ST-segment elevation myocardial infarction. Myocardial enzyme levels were clearly abnormal in 7 patients, coronary artery stenosis was present in 6 patients, and 4 patients underwent coronary stent implantation. The remaining patients received medical management, and 3 patients died (11–22).; a considerable number of patients may have neither cardiac symptoms nor underlying heart disease, and the mortality rate of myocardial infarction caused by paclitaxel is high. The results of this study indicate that for breast cancer patients without a history of heart disease or other exposure experience, a comprehensive clinical assessment of cardiac history, symptoms, risk factors, and physical examination should be performed on all patients. A search of the FAERS database for 94 cases of acute myocardial infarction (AMI) associated with paclitaxel use revealed a positive association between paclitaxel and AMI, with an OR of 2. 06 (95% CI: 1. 89–2. 57). 42 cases (44%) resulted in death or were life-threatening, and 67 cases (71%) were severe (Table 2). Although the sample size is small, the clinical significance of AMI cannot be ignored, given its severity and mortality. The database includes several paclitaxel-induced AMI cases, but no docetaxel-related cases. We report for the first time a case of acute myocardial infarction following docetaxel chemotherapy, hoping to provide reference for clinical practice and supplement relevant databases.

Furthermore, it is noteworthy that in this case, docetaxel was also used in combination with anthracycline cardiotoxic drugs for the treatment of breast cancer, and this combination sequential chemotherapy regimen can also increase drug toxicity (33). Cardiotoxicity is one of the most common adverse reactions in breast cancer patients receiving adjuvant anthracycline therapy, and this issue has been extensively studied in many studies (34–37). Studies by Shah, Kajal et al. have shown that the increased cardiotoxicity risk of paclitaxel and anthracyclines may be related to a pharmacokinetic interaction, namely, cremophor reduces the clearance of anthracyclines in the kidneys and liver, leading to elevated plasma levels (33). Gianni L, Munzone E, et al. have proposed the hypothesis that paclitaxel can stimulate the formation of toxic doxorubicin metabolites in the heart. The combined use of anthracyclines and docetaxel in the treatment of breast cancer may increase the risk of acute myocardial infarction, whether due to the superposition of drug toxicity or pharmacokinetic factors (38). In cancer treatment, the toxicity and allergic reactions of chemotherapy drugs may cause coronary vasoconstriction, increase myocardial oxygen consumption, and simultaneously enhance the body's inflammatory response and hypercoagulable state, thereby accelerating the formation of coronary atherosclerosis, causing endothelial injury and thrombosis. In the study by Yamanaka T et al. endothelin-1 (ET-1) in the vascular endothelium was considered to promote atherosclerosis by facilitating the adhesion of macrophages and other monocytes to endothelial cells and their uptake by endothelial cells, with cardiotoxic chemotherapy potentially further increasing this risk (39),. Studies by Doganci et al. indicate that cytotoxic chemotherapy is associated with an increased risk of vascular thrombotic events, with mechanisms including endothelial injury, reduced levels of anticoagulant factors, elevated levels of procoagulant factors, and the direct toxic effects of chemotherapy drugs on coronary artery endothelium, leading to intimal damage, desquamation, platelet aggregation, and fibrin formation resulting in coronary thrombosis, potentially causing acute myocardial infarction. Related literature reports an incidence of arterial thrombosis in cancer patients of 3. 8% (40). In the cases reported by Feenstra J et al. advanced breast cancer patients developed mesenteric vein thrombosis within one week after first use of docetaxel. Although docetaxel can be considered an important advancement in chemotherapy for several cancers with relatively good cardiovascular safety, ongoing and future trials must assess its role in standardised cancer chemotherapy (41). Clinical treatment requires the use of antithrombotic therapy to reduce cardiovascular risk. There are well-documented cardiovascular adverse reactions associated with docetaxel, both common and rare, which may help comprehensively evaluate the risk-benefit profile of docetaxel.

Cardiac damage caused by breast cancer treatment is becoming increasingly prominent in breast cancer patients. Prevention and treatment of breast cancer-related heart disease has become a new breakthrough in improving the long-term survival rate of breast cancer patients. Systemic inflammatory responses activated by the tumour microenvironment can induce and exacerbate ischemic heart disease, accelerate atherosclerosis, vascular endothelial damage, and thrombosis risk, all of which increase the risk of myocardial infarction (MI) (42, 43). The incidence of myocardial infarction depends on the choice of chemotherapy drugs, potential comorbidities, HER-2 expression, treatment duration, and risk factors of susceptible populations. These factors lead to additive or even synergistic effects, thereby increasing the risk of myocardial infarction. Related studies have proposed relevant indicators that can predict cardiotoxicity at an early stage (36, 44). Biomarkers, including five miRNAs, high-sensitivity troponin, NT- proBNP, and hemoglobin and blood iron content, which are the most readily obtainable in clinical practice, have the potential to predict chemotherapy-induced cardiotoxicity in breast cancer and can be considered early predictors and prognostic markers,ROC curve displayed good predictive value of combining miR-17-5p and miR-20a for lower cardiotoxicity risk (35, 36). Fujii T et al. showed that pegastatin may reduce chemotherapy-induced cardiotoxicity (45). In the study by Moon, Chulso et al. docetaxel may be an alternative treatment for patients allergic to paclitaxel-induced cardiotoxicity (46). In this case, docetaxel can also cause severe myocardial infarction, and this alternative treatment should be approached with caution. Studies on continuous cardiotoxicity monitoring during chemotherapy have shown that routine cardiac monitoring is not necessary for patients without risk factors (23). However, in this case, even for low-risk patients without related heart disease, it is recommended to perform basic cardiac function monitoring and pay attention to the possibility of any fatal cardiotoxic adverse events during any chemotherapy regimen.
